# Exact transcript quantification over splice graphs

**DOI:** 10.1186/s13015-021-00184-7

**Published:** 2021-05-10

**Authors:** Cong Ma, Hongyu Zheng, Carl Kingsford

**Affiliations:** 1 Computational Biology Department, School of Computer Science, Carnegie Mellon University, 5000 Forbes Avenue, Pittsburgh, PA 15213 USA; 2 Computer Science Department, School of Engineering and Applied Science, Princeton University, 35 Olden Street, Princeton, NJ 08544 USA

**Keywords:** RNA-seq, Alternative splicing, Transcript quantification, Splice graph, Network flow

## Abstract

**Background:**

The probability of sequencing a set of RNA-seq reads can be directly modeled using the abundances of splice junctions in splice graphs instead of the abundances of a list of transcripts. We call this model graph quantification, which was first proposed by Bernard et al. (Bioinformatics 30:2447–55, 2014). The model can be viewed as a generalization of transcript expression quantification where every full path in the splice graph is a possible transcript. However, the previous graph quantification model assumes the length of single-end reads or paired-end fragments is fixed.

**Results:**

We provide an improvement of this model to handle variable-length reads or fragments and incorporate bias correction. We prove that our model is equivalent to running a transcript quantifier with exactly the set of all compatible transcripts. The key to our method is constructing an extension of the splice graph based on Aho-Corasick automata. The proof of equivalence is based on a novel reparameterization of the read generation model of a state-of-art transcript quantification method.

**Conclusion:**

We propose a new approach for graph quantification, which is useful for modeling scenarios where reference transcriptome is incomplete or not available and can be further used in transcriptome assembly or alternative splicing analysis.

**Supplementary Information:**

The online version contains supplementary material available at 10.1186/s13015-021-00184-7.

## Background

Transcript quantification has been a key component of RNA-seq analysis pipelines, and the most popular approaches (such as RSEM [1], kallisto [2], and Salmon [3]) estimate the abundance of individual transcripts by inference over a generative model from transcripts to observed reads. To generate a read in the model, a transcript is first sampled proportional to its relative abundance multiplied by length, then a fragment is sampled as a subsequence of the transcript according to bias correction models. The quantification algorithm thus takes the reference transcriptome and the set of reads as input and outputs a most probable set of relative abundances under the model. We focus on a generalization of the problem, called graph quantification, that allows for better handling of uncertainty in the reference transcriptome.

The concept of graph quantification was first proposed by Bernard et al. [4], which introduced a method called FlipFlop. Instead of a set of linear transcripts, a splice graph is given and every transcript compatible with the splice graph (a path from transcript start to termination in the splice graph) is assumed to be able to express reads. The goal is to infer the abundance of edges of the splice graph (or its extensions) under flow balance constraints. Transcript abundances are obtained by flow decomposition under this setup. FlipFlop infers network flow on its extension of splice graphs, called fragment graphs, and uses the model to further assemble transcripts. However, the proposed fragment graph model only retains its theoretical guarantee when the lengths of single-end reads or paired-end fragments are fixed. In this work, we propose an alternative approach to graph quantification that correctly addresses the variable-length reads and corrects for sequencing biases. Our method is based on flow inference on a different extension of the splice graph.

Modeling RNA-seq reads directly by network flow on splice graphs (or variants) is advantageous when the set of transcript sequences is uncertain or incomplete. It is unlikely that the set of reference transcripts is correct and complete for all genes in all tissues, and therefore, many transcriptome assembly methods have been developed for reconstructing a set of expressed transcripts from RNA-seq data [5–8], including FlipFlop [4]. Recent long-read sequencing confirms the expression of unannotated transcripts [9], but it also shows that the individual exons and splice junctions are relatively accurate. With incomplete reference transcripts but correct splice graphs, it is more appropriate to model RNA-seq reads directly by splice graph network flows compared to modeling using the abundances of an incomplete set of transcripts.

The network flow of graph quantification may be incorporated into other transcriptome assembly methods in addition to FlipFlop. StringTie [6] iteratively finds the heaviest path of a flow network constructed from splice graphs. A theoretical work by Shao et al. [10] studies the minimum path decomposition of splice graphs when the edge abundances satisfy flow balance constraints. Better network flow estimation on splice graphs inspires improvement of transcriptome assembly methods.

The splice graph flow itself is biologically meaningful as it indicates the relative usage of splice junctions. Estimates of these quantities can be used to study alternative splicing patterns under the incomplete reference assumption. PSG [11] pioneered this line of work but with a different abundance representation. It models splice junction usage by fixed-order Markov transition probabilities from one exon (or fixed number of predecessor exons) to its successor exon in the splice graph. It develops a statistical model to detect the difference in transition probability between two groups of samples. However, a fixed-order Markov chain has limitations: a small order cannot capture long-range phasing relationships, and a large order requires inferring a number of transition probabilities that are likely to lack sufficient read support. Markov models set the abundance of a transcript to the product of transition probabilities of its splice junctions, which implicitly places a strong constraint on the resulting transcriptome. Many other previous studies of splice junction usage depend on a list of reference transcripts and compute the widely used metric Percentage Spliced In (PSI) [12–14]. Under an incomplete reference assumption, the estimated network flow is a potential candidate to compute PSI and study alternative splicing usage.

A key challenge of graph quantification, especially for paired-end reads, is to incorporate the co-existence relationship among exons in transcripts. When a read spans multiple exons, the exons must co-exist in the transcript that generates this read. Such a co-existence relationship is called phasing, and the corresponding read is said to contain phasing information. For these reads, the flows of the spanned splice edges may be different from each other, and in this case, the probability of the read cannot be uniquely inferred from the original splice graph flow. FlipFlop solves this problem by expanding the splice graph into a fragment graph, assuming all reads are fixed-length. In a fragment graph, every vertex represents a phasing path, two vertices are connected if the phasing paths represented by the vertices differ by one exon, and every transcript on the splice graph maps to a path on the fragment graph. The mapped path in the fragment graph contains every possible phasing path from a read in the transcript, in ascending order of genomic location. However, it is not possible to construct this expansion of splice graphs when the reads or fragments are of variable lengths. There is no longer a clear total order over all phasing paths possible from a given transcript, and it is unclear how to order the phasing paths in a fragment graph. We detail the FlipFlop model in Additional file 1: Section 1.1.

To incorporate the phasing information from variable-length reads or fragments, we develop a dynamic unrolling technique over the splice graph with an Aho-Corasick automaton. The resulting graph is called a prefix graph. We prove that optimizing a network flow on the prefix graph is equivalent to optimizing abundances of reference transcripts using the state-of-the-art transcript expression quantification formulation when all full paths of splice graphs are provided as reference transcripts, assuming modeled biases of generating a fragment are determined by the fragment sequence itself regardless of which transcript it is from. In other words, quantification on prefix graphs generates exact quantification for the whole set of full splice graph paths. The proof is done by reparameterizing the sequencing read generation model from transcript abundances to edge abundances in the prefix graph. We also propose a specialized EM algorithm to efficiently infer a prefix graph flow that solves the graph quantification problem.

As a case study, we apply our method to paired-end RNA-seq data of bipolar disease samples and estimate flows for neurogenesis-related genes, which are known to have complex alternative splicing patterns and unannotated isoforms. We use this case study to demonstrate the applicability of our method to handle variable-length fragments. Additionally, the network flow leads to different PSI compared to the one computed with reference transcripts, suggesting reference completeness should be considered in alternative splicing analysis.

## Methods

We now provide a brief technical overview of the method section.

In "Reparameterization" section, we describe the detailed derivation and procedure to reparameterize the generative model in transcript quantification. A key component in this process is redefining transcript effective length. The transcript effective length is introduced to offset sampling biases towards shorter transcripts, and an empirical formula penalizing transcript length with average fragment length has been widely used. We show that this empirical formula has a more elegant explanation. From this, we naturally introduce the path abundances, the new set of variables that parameterize the generative model, and the path effective lengths, an analogue to transcript effective length that plays a role in normalization. To introduce bias correction, we introduce the concept of affinity that encodes bias-corrected likelihood for generating a fragment at a particular location, and the rest follows naturally by redefining the effective lengths.

In "Prefix graphs" section, we describe the prefix graph, whose purpose is to map the abundances of compatible transcripts (transcripts that correspond to $S-T$ paths on the splice graph) onto network flows that preserve path abundances. This is beneficial, as we avoid enumerating compatible transcripts and only need to infer the prefix graph flow. The key technical contribution in this section is connecting the process of matching phasing paths onto transcripts to the general problem of multi-pattern matching. This leads to a rollout of the splice graph according to an Aho-Corasick automaton, and the correctness (that the flow preserves path abundances) can be proved by running the Aho-Corasick algorithm on the compatible transcripts.

In "Inference" section, we describe the inference process for the prefix graph flows, as we need to expand our model to handle multi-mapped reads, including reads mapped to different genes. We employ a standard EM algorithm for multi-mapped reads, similar to existing approaches. Inference across genes is enabled by another reparameterization of the generative model, which relativizes edge abundances to its incident gene. We decouple the inference for each gene during the M-step, which combined with a simple E-step, allows for efficient inference and completes the specification of our methods.

We formally define the following terms. A **splice graph** is a directed acyclic graph representing alternative splicing events in a gene. The graph has two special vertices: *S* represents the start of transcripts and *T* represents the termination of transcripts. Every other vertex represents an exon or a partial exon. Edges in the splice graph represent **splice junctions**, potential adjacency between the exons in transcripts, or connect two adjacent partial exons. A **path** is a list of vertices such that every adjacent pair is connected by an edge, and an $S-T$ path is a path that starts with *S* and ends with *T*. Each transcript corresponds to a unique $S-T$ path in the splice graph, and as discussed in the introduction we will assume every $S-T$ path is also a potential transcript. Graph quantification generalizes transcript quantification as we can set up a “fully rolled out” splice graph containing only chains that each corresponding to a linear transcript. We use the phrase **quantified transcript set** to denote a set of transcripts with corresponding abundances.

Finally, we use the term **phasing paths** extensively. In its original definition, phasing paths are derived from reads that span more than two exons. As each read (or read pair) originates from a single transcript, a transcript containing the phasing path must be present in the transcriptome. These paths provide valuable information in determining longer-range exon arrangements. We generalize the notion and remove the constraint that phasing paths must contain more than two exons. Specifically, singleton paths (paths that consisting of a single vertex) are also considered phasing paths. Under this definition, all fragments (mapped from reads) can be mapped to a phasing path.

### Reparameterization

Our goal in this section is to establish an alternative set of parameters for the graph quantification problem. In the transcript quantification model, every transcript corresponds to a variable denoting its relative abundance. We will identify a more compact set of parameters that would represent the same model, as described below.

We start with the core model of transcript quantification at the foundation of most modern methods [1–3, 15]. Assume the paired-end reads from an RNA-seq experiment are error-free and uniquely aligned to a reference genome with possible gaps as fragments (these assumptions will be relaxed later). We denote the set of fragments (mapped from paired-end reads) as *F*, the set of transcripts as ${\mathcal {T}}= \{T_1, T_2, \dots , T_n\}$ with corresponding lengths $l_1, l_2, \dots , l_n$ and abundances (copies of molecules) $c_1, c_2, \dots , c_n$. This can be used to derive other quantities. For example, the transcripts per million (TPM) values are calculated by normalizing $\{c_i\}$ then multiplying the values by $10^6$. Under the core model, the probability of observing *F* is:

$$\begin{aligned} P(F\mid {\mathcal {T}}, c) = \prod _{f\in F} \sum _{i\in \text {idx}(f)} P(T_i) P(f\mid T_i). \end{aligned}$$

Here, $P(T_i)$ denotes the probability of sampling a fragment from transcript $T_i$ and $P(f\mid T_i)$ denotes the probability of sampling the fragment *f* given that it comes from $T_i$. $\text {idx}(f)$ is the set of transcript indices onto which *f* can map. Let *D*(*l*) be the distribution of generated fragment lengths. In the absence of bias correction, $P(f\mid T_i)$ is proportional to $D(f)=D(l(f))$ where *l*(*f*) denotes the fragment length inferred from mapping *f* to $T_i$. Define the effective length for $T_i$ as ${\hat{l}}_i = \sum _{j=1}^{l_i} \sum _{k=j}^{l_i} D(k - j + 1)$ (which can be interpreted as the total “probability” for $T_i$ to generate a fragment), and $P(f\mid T_i) = D(f)/{\hat{l}}_i$. The probability of generating a fragment from $T_i$ is assumed to be proportional to its abundance times its effective length, meaning $P(T_i) \propto c_i{\hat{l}}_i$. Our definition of effective length is different from existing literature, where it is usually defined as $l_i - \mu (T_i)$, the actual length of transcript $l_i$ minus the truncated mean of *D*, and the truncated mean is defined as $\mu (T_i) = (\sum _{j=1}^{l_i} jD(j))/(\sum _{k=1}^{l_i} D(k))$. However, these two definitions are actually essentially the same most of the time.

#### **Lemma 1**

$${\hat{l}}_i = \sum _{j=1}^{l_i} \sum _{k=j}^{l_i} D(k - j + 1) = (\sum _{t=1}^{l_i} D(t))(l_i + 1 - \mu (T_i))$$.

#### *Proof*

$$\begin{aligned} {\hat{l}}_i&= \sum _{t=1}^{l_i} D(t)(l_i+1-t) \\&= (l_i + 1) \sum _{t=1}^{l_i} D(t) - \sum _{t=1}^{l_i} tD(t) \\&= \left( \sum _{t=1}^{l_i} D(t)\right) \left( l_i + 1 - \frac{\sum _{t=1}^{l_i} tD(t)}{\sum _{t=1}^{l_i} D(t)}\right) \\&= \left( \sum _{t=1}^{l_i} D(t)\right) \left( l_i + 1 - \mu (T_i)\right) \end{aligned}$$

This means ignoring the difference between $l_i$ and $l_i+1$, the two definitions differ by a multiplicative factor of $\sum _{t=1}^{l_i} D(t)$. $\square$

The factor $\sum _{t=1}^{l_i} D(t)$ is the probability of sampling a fragment no longer than $l_i$. It is very close to 1 as long as the transcript is longer than most fragments, which is usually true in practice. We refer to previous papers [1–3, 15, 16] for more detailed explanation of the model. This leads to:

$$\begin{aligned} P(F\mid {\mathcal {T}}, c) = \prod _{f\in F} \left( \sum _{i\in \text {idx}(f)} c_i\right) D(f) / (\sum _{T_i \in {\mathcal {T}}} c_i {\hat{l}}_i). \end{aligned}$$

We now propose an alternative view of the probabilistic model with paths on splice graphs to derive a compact parameter set for the quantification problem. The splice graph is constructed so that each transcript can be uniquely mapped to an $S-T$ path $p(T_i)$ on the graph, and we assume the read library allows each fragment *f* to be uniquely mapped to a (non $S-T$) path *p*(*f*) on the graph (this assumption will also be relaxed later). With this setup, $i\in \text {idx}(f)$ if and only if *p*(*f*) is a subpath of $p(T_i)$ (in other words, $p(f) \subset p(T_i)$).

We now define $c_p = \sum _{i: T_i \in {\mathcal {T}}, p\subset p(T_i)} c_i$ to be the total abundance of transcripts including path *p*, called **path abundance**, and ${\hat{l}}_p = \sum _{j=1}^{l_i} \sum _{k=j}^{l_i} {\mathbf {1}}(p(T_i[j,k]) = p)D(k-j+1)$ called **path effective length**, where $T_i[j, k]$ is the fragment generated from transcript *i* from base *j* to base *k* and $\mathbf{1} (\cdot )$ is the indicator function. Intuitively, the path effective length is the total probability of sampling a fragment that maps exactly to the given path. This definition is independent of the chosen transcript $T_i$ and any $T_i$ yields the same result as long as $T_i$ includes *p*. Next, let ${\mathcal {P}}$ be the set of paths from the splice graph satisfying ${\hat{l}}_p > 0$.

#### **Lemma 2**

*The normalization term can be reparameterized*: $\sum _{T_i \in {\mathcal {T}}} c_i {\hat{l}}_i = \sum _{p\in {\mathcal {P}}} c_p {\hat{l}}_p$.

#### *Proof*

The idea is to break down the expression of ${\hat{l}}_i$ into a sum over fragments, and regroup the fragments by the path to which they are mapped:

$$\begin{aligned} \sum _{T_i\in {\mathcal {T}}} {\hat{l}}_i c_i&= \sum _{T_i \in {\mathcal {T}}} \sum _{j=1}^{l_i} \sum _{k=j}^{l_i} D(k-j+1)c_i \\&= \sum _{p\in {\mathcal {P}}} \sum _{i, j, k: p(T_i[j, k]) = p} D(k-j+1)c_i \\&= \sum _{p\in {\mathcal {P}}} \left( \sum _{j, k: \exists i, p(T_i[j, k]) = p} D(k - j+1)\right) \left( \sum _{i: p\subset p(T_i)} c_i\right) \\&= \sum _{p\in {\mathcal {P}}} {\hat{l}}_p c_p. \end{aligned}$$

The third equation holds because the sum of $D(k-j+1)$ across any transcripts containing path *p* is the same, as a shift in the reference does not change $D(k-j+1)$ assuming there are no sequencing biases. $\square$

The likelihood objective can now be rewritten as follows:

1 $$\begin{aligned} \begin{aligned} P(F\mid {\mathcal {T}}, c)& = \prod _{f\in F} \left( \sum _{j: p(f)\subset p(T_j)} c_j\right) D(f) / \left(\sum _{p\in {\mathcal {P}}} c_p {\hat{l}}_p \right) \\& \quad \propto \prod _{f\in F} c_{p(f)} / \left( \sum _{p\in {\mathcal {P}}} c_p {\hat{l}}_p\right) . \end{aligned} \end{aligned}$$

This reparameterizes the model with $\{c_p\}$, the path abundance. To incorporate bias correction into our model, we define the affinity $A_p(j, k)$ to be the unnormalized likelihood of generating a read pair mapped to path *p* from position *j* to *k*. This is the analog of $P(f\mid t_i)$ in the transcript quantification model. In the non-bias-corrected model, we simply have $A_p(j, k) = D(k-j+1)$. Certain motif-based corrections and GC-content-based corrections, which are calculated from the genomic sequence in between the paired-end alignment, can then be integrated into our analysis naturally. To adapt the likelihood model to bias correction, we define transcript and path effective length as follows:

$$\begin{aligned} {\hat{l}}_i&= \sum _{j=1}^{l_i} \sum _{k=j}^{l_i} A_{p(T_i[j,k])}(j,k) \\ {\hat{l}}_p&= \sum _{j=1}^{l_i} \sum _{k=j}^{l_i} A_p(j, k)\mathbf{1} (p(T_i[j,k])=p),\quad \forall p\subset p(T_i) \end{aligned}$$

${\hat{l}}_p$ is still the same for any $T_i$ that includes *p*, so it does not matter which transcript is used to compute it. $p(T_i[j, k])$ denotes the path that $T_i[j, k]$ (transcript $T_i$ from location *j* to *k*) maps to, and we assume the coordinate when calculating $A_p$ coincides with that of $T_i$. The definition of path abundance remains unchanged, and all of our proposed methods will work in the same way. Transcript-specific bias correction requires an approximation to the affinity term, and we discuss this topic in detail later in this section.

We have now completed the necessary steps to claim the following theorem, which formally establishes the correctness of the reparameterization procedure with bias correction.

#### **Theorem 1**

*Assuming each read is uniquely mapped to one phasing path, the following two optimization instances are equivalent:*

- *Optimizing* $\{c_p\}$, *which are the path abundances under the reparameterized objective* $\prod _{f\in F} c_{p(f)} / (\sum _{p\in {\mathcal {P}}} c_p {\hat{l}}_p)$*, conditioned on* $\{c_p\}$* corresponding to a valid quantified set of transcripts;*
- *Optimizing* $\{c_i\}$* which are the transcript abundances under the original objective* $\prod _{f\in F} (\sum _{i\in \text {idx}(f)} c_i) / (\sum _{T_i \in {\mathcal {T}}}c_i{\hat{l}}_i)$.

*Here* ${\hat{l}}_i$ *and* ${\hat{l}}_p$ *are transcript effective length and path effective length defined with the same set of affinities* $A_p(j, k)$.

#### *Proof*

This naturally follows in two steps. First, we can prove Lemma 2 with bias correction using the identical technique of breaking ${\hat{l}}_i$ down to sum over fragments, then regroup by path mappings. This means the normalization term can be reparameterized. We finish by reparameterizing the whole likelihood in the same way as in the non-bias-corrected case (see Eq. (1)), again with the identical technique. $\square$

With reads multimapped to different phasing paths (within or across genes), let *M*(*f*) denote the set of phasing paths *f* can map onto, and for $p \in M(f)$ let $A(f\mid p)$ denote the affinity of *f* mapping to *p*. In this case, we can use the same idea of grouping transcripts by the phasing path that *f* maps onto:

$$\begin{aligned} P(f)&= \sum _{i\in \text {idx}(f)} P(T_i)P(f\mid T_i)\\&= \sum _{p\in M(f)} \sum _{i: p\subset T_i} c_i A(f\mid p) \\&= \sum _{p\in M(f)} c_pA(f\mid p). \end{aligned}$$

The reparameterization theorem holds by replacing $c_{p(f)}$ with $\sum _{p\in M(f)} c_pA(f\mid p)$ in the objective function.

### Prefix graphs

#### Theoretical foundation of prefix graphs

In Theorem 1, we showed that to perform graph quantification, it is sufficient to optimize the path abundances under a reparameterized objective, requiring that the path abundances correspond to a quantified set of transcripts. This means that to apply the theorem for optimization of path abundance, we need a set of constraints that ensures this condition. One solution is to introduce a variable for every compatible transcript and then use the definition of $c_p$ as the constraints. However, this will lead to an impractically large model, as the number of $S-T$ paths in the splice graph can be exponentially larger than the size of the prefix graph. In this section, we derive a set of linear constraints governing $\{c_p\}$ that achieves this purpose.

To motivate the next step, assume every inferred fragment either resides within an exon or contains one junction. In this case, the phasing paths are nodes or edges in the splice graph. More specifically, when the fragment resides within an exon, the phasing path contains that exon only, and when the fragment contains one junction the phasing path contains the two constituent exons that form an edge in the splice graph. If the quantified transcript set is mapped onto the splice graph, we obtain a network flow. The path abundance for a phasing path equals either the flow through a vertex or an edge. By the flow decomposition theorem (that every network flow over a DAG can be decomposed into finitely many paths), given a network flow on the splice graph, we can decompose it into $S-T$ paths with weights. These paths naturally map back to a quantified transcript set. As the two-way mapping (between quantified transcript sets and splice graph flows) preserves path abundances, we conclude optimization over a splice graph flow would achieve the goal of graph quantification. Specifically, it is easy to restructure the constraints to represent a splice graph flow, and optimizing the resulting model is equivalent to the transcript quantification model with all compatible transcripts included.

This solution no longer works when some phasing path *p* contains three or more exons. This is because one cannot determine the total flow that goes through two consecutive edges (corresponding to a phasing path with two junctions) just from the flow graph, and different decompositions of the flow can lead to different answers. Informally, this can be solved by constructing higher-order splice graphs (as done by Legault et al. [11] for example), or fixed-order Markov models, but the size of the resulting graph grows exponentially fast and some phasing paths can be very long. Instead, we choose to “unroll” the graph just as needed, roughly corresponding to a variable-order Markov model, similar to FlipFlop [4] but applicable to variable-length paired-end reads.

To motivate our proposed unrolling method, consider the properties it needs to satisfy. Roughly speaking, the unrolled graph needs to exactly identify every path in ${\mathcal {P}}$ to accurately calculate the path abundances. That is, for every path *p* in ${\mathcal {P}}$, there is a set of vertices or edges in the unrolled graph, such that a transcript includes *p* if and only if its corresponding $S-T$ path intersects with this set. We can view this “identify phasing paths” problem as an instance of multiple pattern matching. That is, given ${\mathcal {P}}$, for a given transcript $T_i$, we want to determine the set of paths in ${\mathcal {P}}$ that are subpaths of $T_i$, reading one exon of $T_i$ at a time. Similar to our previous example, if ${\mathcal {P}}$ contains only single exons, we only need to recognize [*x*] (the singleton path including only *x*) when we read exon *x*, and we will recognize a general phasing path *p* when the transcript we have seen admits *p* as a suffix. To speed up the process, we can memorize a suffix of the transcript we have seen that is a prefix of some path in *p*, so we do not need to check all preceding exons again when trying to recognize *p*. This is not a new idea and in fact is the Aho-Corasick algorithm [17], a classical algorithm for multiple pattern matching where the set of nodes in the splice graph (set of exons) is the alphabet, ${\mathcal {P}}$ is the set of patterns and $T_i$ is the text, and the idea is formalized as a finite state automaton (FSA) that maintains the longest suffix of current text that could extend and match a pattern in the future. This can be regarded as an unrolling of the splice graph, which has the power of exactly matching arbitrarily phasing paths, and a flow on the automaton is the analog of a splice graph flow that also is unrolled enough to recover path abundances, as we will prove in this section.

We formalize the idea. Consider the Aho-Corasick FSA constructed from ${\mathcal {P}}$, where we further modify the finite state automaton as follows. Transitions between states of the FSA, called dictionary suffix links, indicate the next state of the FSA given the current state and the upcoming character. We do not need the links for all characters (exons), as we know $T_i \in {\mathcal {T}}$ is an $S-T$ path on the splice graph. If *x* is the last seen character, the next character *y* must be a successor of *x* in the splice graph, and we only generate the state transitions for this set of movements. With an FSA, we now construct a directed graph from its states and transitions as described above:

##### **Definition 1**

*(Prefix Graph)* Given splice graph $G_S$ and set of splice graph paths ${\mathcal {P}}$ (assuming every single-vertex path is in ${\mathcal {P}}$), we construct the corresponding prefix graph $G=(V, E)$ as follows:

The vertices *V* are the splice graph paths *p* such that *p* is a prefix of some path in ${\mathcal {P}}$. For $p \in V$, let *x* be the last exon in *p*. For every *y* that is a successor of *x* in the splice graph, let $p'$ be the longest path in *V* that is a suffix of *py* (*py* is the path generated by appending *y* to *p*). We then add an edge from *p* to $p'$ to *E*.

The source and sink of *G* are the vertices corresponding to splice graph paths [*S*] and [*T*], where [*x*] denotes a single-vertex path. The set *AS*(*p*) is the set of vertices $p'$ such that *p* is a suffix of $p'$.

**Fig. 1:**
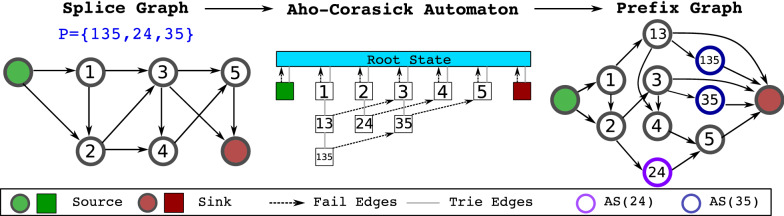
An example construction of the Prefix Graph. The source and sink of the prefix graph are [*S*] and [*T*], respectively. The set of phasing paths ${\mathcal {P}}$ is shown in blue in the left panel, and we do not include the singleton paths for simplicity. We draw the trie and the fail edges for the **a**–**c** automaton as it reduces cluttering (dictionary suffix link can be derived from both edge sets). The colored nodes in prefix graph are the vertices (states) in *AS*(35) and *AS*(24)

Figure 1 shows an example construction of prefix graph. Intuitively, the states of the automaton are the vertices of the graph and are labeled with the suffix in consideration at that state. The edges of the graph are the dictionary suffix links of the FSA, now connecting vertices. For $p \in {\mathcal {P}}$, *AS*(*p*) denotes the set of states in FSA that recognizes *p*. All transcripts start with *S*, end with *T*, and there is no path in ${\mathcal {P}}$ containing either of them as they are not real exons, so there exist two vertices labeled [*S*] and [*T*]. We call them the source and sink of the prefix graph, respectively, and we will see they indeed serve a similar purpose.

##### **Lemma 3**

*There is a one-to-one correspondence between* $S-T$ *paths in the splice graph and* $[S]-[T]$ *paths in the prefix graph.*

##### *Proof*

Every transcript can be mapped to an $[S]-[T]$ path on the prefix graph by feeding the transcript to the finite state automaton and recording the set of visited states, excluding the initial state where no string is matched. The first state after the initial state is always [*S*] as the first vertex in an $S-T$ path is *S*, and the last state is always [*T*] because there are no other vertexes in the prefix graph that would contain *T*. Conversely, a $[S]-[T]$ path on the prefix graph can also be mapped back to a transcript, as it has to follow dictionary suffix links (transitions between FSA states), which by our construction can be mapped back to edges in the splice graph. $\square$

This implies that the prefix graph is also a DAG: If there is a cycle in the prefix graph, it implies an exon appears twice in a transcript, which violates our assumption that the splice graph is a DAG.

The resulting prefix graph flow serves as a bridge between the path abundance $\{c_p\}$ and the quantified transcript set $\{c_i\}$:

##### **Theorem 2**

*Every quantified transcript set can be mapped to and from a prefix graph flow. The path abundance is preserved during the mapping and can be calculated exactly from prefix graph flow*: $c_p = \sum _{s\in AS(p)} f_s$, *where* $f_s$ *is the flow through vertex* *s*.

##### *Proof*

Using the path mapping between the splice graph and the prefix graph, we can map a quantified transcript set onto the prefix graph as a prefix graph flow and reconstruct a quantified transcript set by decomposing the flow and mapping each $[S]-[T]$ path back to the splice graph as a transcript.

To prove the second part, let $\{c_p\}$ be the path abundance calculated from the definition given a quantified transcript set, and $\{c'_p\}$ be the path abundance calculated from the prefix graph flow. We will show $\{c_p\} = \{c'_p\}$ for any finite decomposition of the prefix graph flow.

For any transcript $T_i$ and any path $p \in {\mathcal {P}}$, since no exon appears twice for a transcript, if $T_i$ contains *p*, it will be recognized by the FSA exactly once. This means the $[S]-[T]$ path to which $T_i$ maps intersects with *AS*(*p*) by exactly one vertex in this scenario, and it contributes the same abundance to $c'_p$ and $c_p$. If $T_i$ does not contain *p*, by similar reasoning, it contributes to neither $c'_p$ nor $c_p$. This holds for any transcript and any path, so the two definitions of path abundance coincide and are preserved in mapping from quantified transcript set to prefix graph flow. Since the prefix graph flow is preserved in flow decomposition, the path abundance is preserved as a function of prefix graph flow. $\square$

This connection allows us to directly optimize over $\{c_p\}$ by using the prefix graph flow as variables (the path abundances $c_p$ is now represented as seen in Theorem 2), and use flow balance and non-negativity as constraints, as we describe in the next section. The corresponding quantified transcript set is guaranteed to exist by a flow decomposition followed by the mapping process.

#### Compact prefix graph

We next describe an improvement to the prefix graph, which we call the compact prefix graph. In the prefix graph, FSA states are vertices of the resulting graph, and as *AS*(*p*) is a set of vertices, we can say that we recognize phasing paths at the vertices of the prefix graph. The idea for compact prefix graph is to recognize phasing paths at the edges instead. We will still start with the Aho-Corasick FSA, but we will be building the graph in a way that the states of the FSA correspond to edges of the resulting graph, as described below:

##### **Definition 2**

*(Compact Prefix Graph)* Given splice graph $G_S$ and set of splice graph paths ${\mathcal {P}}$, we construct the corresponding compact prefix graph $G'$ as follows. The vertex set of the compact prefix graph is the union of

- All single-vertex paths on the splice graph;
- Any splice graph path *p* that is the prefix of some path $p'$ in ${\mathcal {P}}$, while being strictly shorter than $p'$.

For *p* in the compact prefix graph, let *x* be its last exon and *y* be a successor of *x* in the splice graph. We create an edge that has label *py* (again, appending *y* to *p*), originates from *p*, and leads to the node that is the longest suffix of *py* in the compact prefix graph.

The source and sink of *G* are the vertices corresponding to splice graph paths [*S*] and [*T*]. The set *AS*(*p*) is the set of edges $p'$ such that the edge label on $p'$ is a suffix of *p*.

The set *AS*(*p*) bears the same meaning as in the original prefix graph, as the states of the Aho-Corasick FSA are now (roughly) the edges of the compact prefix graph. With this intuition, we can prove the same property as stated in Lemma 3 for compact prefix graph.

##### **Lemma 4**

*There is a one-to-one correspondence between* $S-T$ *paths in the splice graph and* $[S]-[T]$ *paths in the compact prefix graph.*

##### *Proof*

Every transcript can be mapped to an $[S]-[T]$ path on the compact prefix graph by walking on the compact prefix graph starting from [*S*]. Upon reading a new exon *y*, we move to the new vertex on the compact prefix graph by following the edge that has a label ending with *y*. Our construction ensures this edge exists and is unique. Similar to our previous reasoning, the finishing state is always [*T*] because there are no other vertexes in the compact prefix graph whose label contains *T*. Conversely, a $[S]-[T]$ path on the prefix graph can also be mapped back to a transcript, by mapping an edge with label *py* on compact prefix graph to an edge (*x*, *y*) on the splice graph, where *x* is the last exon of p. $\square$

With this, we can prove Theorem 3, an analogue of Theorem 2 for compact prefix graphs.

##### **Theorem 3**

*Every quantified transcript set can be mapped to and from a compact prefix graph flow. The path abundance is preserved during the mapping and can be calculated exactly from compact prefix graph flow*: $c_p = \sum _{e\in AS(p)} f_e$, *where* $f_e$ *is the flow through edge* *e*.

The proof is mostly identical to that of Theorem 2 with a minor technical difference. When a phasing path is mapped to a prefix graph edge, the label on that edge might not equal the current FSA state, which is guaranteed in the non-compact prefix graph case. Instead, the current FSA state is a suffix of the edge label. The compact prefix graph, by the virtue of its construction, is smaller compared to the original prefix graph with the same power and is preferred in practice. It also has the desirable property of being a “minimal sufficient” graph in many cases, as we will show below.

##### **Theorem 4**

(Compact Prefix Graph Flow can be Minimally Sufficient) *With fixed splice graph* *G* *and phasing path set* ${\mathcal {P}}$, *if they satisfy the conditions that (1) for each* $p\in {\mathcal {P}}$ *any prefix of* *p* *is also in* ${\mathcal {P}}$ *and (2) each vertex in* *G* *has maximum outgoing degree of 2, there is a one-to-one mapping between the feasible set of path abundance* $\{c_p \mid p\in {\mathcal {P}}\}$ *and the feasible set of compact prefix graph flows* $\{f_e\}$.

##### *Proof*

The mapping from a compact prefix graph flow to $\{c_p\}$ is direct and unique. For the other direction, assuming the theorem is false, there exist two sets of compact prefix graph flow $\{f'_e\}$ and $\{f''_e\}$ with identical path abundances $\{c_p\}$. Since each $c_p$ is the sum of several flow values, the difference between the sets $\{\Delta f_e\}$ (where $\Delta f_e = f'_e - f''_e$ for all edges *e* in the compact prefix graph) satisfies the following:

$$\begin{aligned} \sum _{e\in AS(p)} \Delta f_e = 0, \quad \forall p\in {\mathcal {P}}. \end{aligned}$$

We now prove that $\Delta f_e = 0$ for all *e*, using induction on the size of |*t*(*e*)| (the number of exons in the edge label of *e*), which we denote *k*. The induction hypothesis for *k* is that $\Delta f_e = 0$ for all edges *e* with label no shorter than *k*. For the base case where $k > \max _{e} |t(e)|$, there are no edges with label length *k* or longer, so the hypothesis holds trivially. Here, we use *t*(*e*) to denote the label on an edge in the compact prefix graph, and *t*(*v*) to denote the label on a vertex (the splice graph path it represents) in the compact prefix graph.

Assume this holds for $k + 1$. For each edge $e'$ with label length *k*, let $p'=t(e')$, and we write $p'=px$. By the construction of the compact prefix graph, *p* is a strict prefix of some phasing paths in ${\mathcal {P}}$. We now discuss two cases.

- If $p'$ is also in ${\mathcal {P}}$, there is an equation of form $\sum _{e\in AS(p')} \Delta f_e = 0$. The set $AS(p')$ contains the edge $e'$ and several other edges with edge labels longer than $p'$ (as $p'$ is a suffix of these labels). By the induction hypothesis, $\Delta f_e$ for all edges in $AS(p')$ other than $e'$ equals 0, which means $\Delta f_{e'}$ is also 0.
- If $p' \notin {\mathcal {P}}$, we first claim that the last exon of *p* (second-to-last exon of $p'$) has two outgoing edges. Otherwise, because there are no other phasing paths $p''$ with *p* as a strict prefix, *p* cannot be a vertex of the compact prefix graph; contradiction. We now let *y* be the destination of the other outgoing edge of the last exon of *p*. By similar reasoning, $py \in {\mathcal {P}}$, otherwise *p* cannot be a vertex of the compact prefix graph. With a slight abuse of notion, we let $c_{px}$ denote the total flow of edges whose label admits *px* as a suffix. We claim $c_{px} = c_p - c_{py}$. Intuitively, the total abundance of transcripts containing *px* equals the abundance of transcripts containing *p*, minus those containing *py*, because all transcripts are $S-T$ paths in the splice graph and *px* is the only other way *p* can extend. Formally, for every edge (*u*, *v*) counting towards $c_{px}$, by the construction of compact prefix graph, *t*(*u*) has *p* as a suffix, meaning *u* has exactly one other outgoing edge that counts towards $c_{py}$. This means $c_{px} + c_{py}$ equals the total outgoing flow of all vertexes *u* whose label ends with *p*. On the other hand, for every edge $e'' = (u', v')$ counting towards $c_p$, $t(e'')$ takes *p* as a suffix. Since $p\in {\mathcal {P}}$ and $v'$ is the longest suffix of $t(e'')$ in ${\mathcal {P}}$, we conclude $t(v')$ also has *p* as a suffix. Those edges such that *p* is a suffix of $t(v')$ also count towards $c_p$ by definition. We conclude that $c_p$ equals the total incoming flow of all vertexes *v* whose label ends with *p*. By the flow balance condition, $c_p = c_{px} + c_{py}$. Finally, since $c_p$ is the same between two sets of $\{f_e\}$, and $c_{py}$ is the same between them too, $c_{px}$ must also be the same. We can then use the argument from the first case as if $px \in {\mathcal {P}}$, and conclude $\Delta f_{e'} = 0$.

In both cases we conclude $\Delta f_{e'} = 0$ for all edges $e'$ satisfying $|t(e')| = k$, which completes the induction proof. $\square$

In other words, the unrolling of the splice graph by constructing a compact prefix graph is, in a certain sense, the optimal unrolling. This theorem is also very conservative, and both of its requirements are not necessary for the uniqueness condition to hold.

#### Practical considerations of prefix graphs

We discuss three changes to the prefix graph framework when we implement it for the analysis of real RNA-seq data. These changes are necessary to enable practical analysis under our proposed framework, with minimal changes to the theoretical foundation.

*Approximating Transcript-Specific Bias Correction.* Positional bias is a common factor taken into consideration by modern transcript quantification methods. However, they cannot be integrated into our proposed model of graph quantification directly, because the positional bias term would change depending on every exon in the transcript, not just those in a phasing path. Nonetheless, since the splice graph is known in full, approximating positional bias is possible.

Let $B_i(j, k)$ denote the affinity value calculated from a full bias correction model for the fragment generated from base *j* to *k* on transcript $T_i$, and ${\hat{r}}_i$ be the reference abundance of transcript $T_i$. We let $A_p(j, k) = (\sum _{i: p\subset p(T_i)} {\hat{r}}_i B_i(j', k')) / (\sum _{i:p\subset p(T_i)} {\hat{r}}_i)$, where $j'$ and $k'$ are the coordinates of the path sequence in the reference coordinates of $T_i$. In other words, for each phasing path, we calculate the empirical positional bias, collected over reference transcriptome and weighted with reference abundances.

In our experiments, for simplicity we use Salmon outputs as reference abundance and calculate positional bias accordingly, using the same positional bias model as Salmon. It is also possible to model the estimation of positional bias terms as an iterative process where we alternatively estimate the path abundances $c_p$ and the bias correction terms $A_p(j, k)$.

*Calculation of Path Effective Length: Switching Affinities.* We have derived a closed and explicit form for ${\hat{l}}_p$, the path effective length, which can be calculated given the affinity values $A_p(j, k)$ with bias correction. In practice, the value of $A_p(j, k)$ comes from complicated models of biases. We can speed up the calculation of ${\hat{l}}_p$ by reusing calculated affinities, however, to obtain the correct value of path effective length for each phasing path, we need to calculate the affinities for every possible fragment.

For paired-end sequencing, while the reads are of fixed length and short, the induced fragment length can vary in a wide range. If the longest fragments are of length *M* and the reference sequence has length *N*, the number of affinity values required may reach *O*(*MN*). While this bound is not reached in practice, the process of exact calculation of path effective length is slow.

While we decided to calculate these exactly for our experiments, there are possible approximations to avoid this time-consuming step. Existing methods avoid this by essentially using two sets of affinity values, in calculating transcript effective length ${\hat{l}}_i$ and calculating read likelihood $P(f\mid T_i)$. To see why this is the case, Lemma 1 indicates that existing methods to calculate the effective length of a transcript are approximately summing up affinity values from fragments generated on this transcript before bias correction. In other words, the non-bias-corrected affinity values are used in calculating ${\hat{l}}_i$. However, when calculating read likelihood, the bias-corrected affinity values are used instead. It is an interesting open question that whether “correcting” this discrepancy in existing methods for transcript quantification, by calculating transcript effective length in the “correct way” of summing up bias-corrected affinity values, would lead to better results.

For our proposed graph quantification methods, the aforementioned “two affinities” approximation is a justifiable route, as the path effective length ${\hat{l}}_p$ also has a closed-form solution without bias correction. Recall this means $A_p(j, k) = D(k-j+1)$, and each ${\hat{l}}_p$ is weighted sum of some *D*(*i*). This can be evaluated in constant time with proper preprocessing. Alternatively, for multiple RNA-seq experiments sharing the same set of splice graphs, we propose to use the idea of “two affinities” in a more refined way, with two bias-corrected models. We can first derive a common ground bias correction model shared across all experiments, then calculate the exact path effective length using the shared bias model. This is costly but only needs to be done once. The fragment likelihood $P(f\mid T_i)$ would then be calculated using the bias model private to each experiment. We believe the integration of proper bias correction during calculation of path effective length would improve the practicality of graph quantification models.

*Trimming *${\mathcal {P}}$,*the set of phasing paths.* For simplicity, we for now consider graph quantification without bias correction, meaning $A_p(j, k) = D(k-j+1)$. Recall the original definition of ${\mathcal {P}}$: It is the set of all phasing paths that have a non-zero path effective length. In practice, the fragment length distribution $D(\cdot )$ has a long tail. Using *M* to denote the longest fragment that can be generated from *D*, ${\mathcal {P}}$ would include the set of phasing paths that can generate a fragment no longer than *M*. In practice, such a long tail results in a huge ${\mathcal {P}}$. This is problematic, losing the benefits of using graph quantification in theory and slowing the inference in practice. We trim ${\mathcal {P}}$ by removing phasing paths with small effective lengths and no mapped fragments. The removed paths are usually those extra-long phasing paths that only can generate a fragment thanks to the long tail of the fragment length distribution. As longer phasing paths involve more complex splicing patterns, the numbers of such paths are large, and the trimming is highly effective in practice.

To further justify our choice more rigorously, we recall the likelihood function under our reparameterized model: $P(F\mid {\mathcal {T}}, c) \propto \prod _{f\in F} c_{p(f)} / (\sum _{p\in {\mathcal {P}}} c_p {\hat{l}}_p)$. Now, we investigate the effect of phasing paths $p'$ without mapped fragments on the likelihood. These phasing paths do not contribute to the fragment likelihood term because there are no fragments $f\in F$ such that $p(f)=p'$. However, their abundance contributes towards the normalization term. This means if we ignore the phasing paths without mapped fragments, we underestimate the normalization term during optimization, and in turn, overestimate transcript and path abundances. We argue the magnitude of overestimation is small. As we are only removing phasing paths with small ${\hat{l}}_p$, the only way this changes our results significantly is that some of these paths have large estimated $c_p$. If there is a removed path with a large $c_p$ when optimized under this model, it means in the inferred quantified transcript set there is are many fragments mapped to path *p*, even though exactly zero fragments are mapped to *p* in the sequencing library. This mostly happens if there is a dominant transcript with high abundance, and *p* is part of the transcript. For such things to happen, the transcript must have many fragments mappable, and the fact that no single fragment mapped to path *p* indicates ${\hat{l}}_p$ is small, or the modeling might be faulty.

### Inference

While the restructuring process described in the previous section reduces the size of the optimization problem, we still need to solve it efficiently. We start with the base case, that is, the genome contains a single gene, and every read pair maps to exactly one path. Recall that ${\mathcal {P}}$ is the set of phasing paths we consider, *p*(*f*) is the path fragment *f* maps to. For a phasing path *p*, $c_p$ is the path abundance, ${\hat{l}}_p$ is the path effective length. For the prefix graph, we let $f_e$ denote the flow through an edge, $f_v$ denote the flow through a vertex, and *AS*(*p*) is the set of vertices (as FSA states) that recognize *p*. We also use $\text {In}(v)$ to denote the incoming edges of vertex *v*, and $\text {Out}(v)$ similarly for the outgoing edges. The full instance in this case, with the prefix graph proposed the previous section, is:
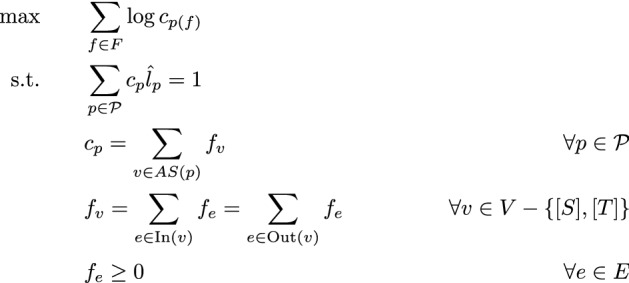


This is slightly different from what we described in "Reparameterization" section. First, we maximize the logarithm of the likelihood objective. Second, we explicitly fix the normalization constant to be 1, instead of placing it on the divisor of the fragment likelihood. This does not change the objective, and the only difference is that in the original form $\{c_p\}$ can be arbitrarily scaled, while here the scaling is fixed. The variables and the constraints come from the prefix graph flow, and $c_p$ is represented as in Theorems 2 and 3. This is a convex problem, as the target function is convex with respect to $\{c_p\}$, and the constraints are all linear. We can solve the problem with general-purpose convex solvers.

With the presence of multi-mapped reads (to multiple genes and/or multiple paths within one gene), we can employ a standard EM approach. Recall *M*(*f*) is the set of phasing paths onto which *f* can map, and for $p \in M(f)$ let $A(f\mid p)$ denote the affinity of *f* mapping to *p*, as described in Section 2.1. We also let *z* denote the hidden allocation vector, where $z_{f, p}$ denotes the probability that the fragment *f* is mapped onto splice graph path *p*. We can alternatively optimize for $\{z_{f, p}\}$ and $\{c_p\}$ until convergence as follows:

$$\begin{aligned} z^{(t)}_{f, p}&= c^{(t)}_pA(f\mid p) / \left( \sum _{p'\in M(f)} c^{(t)}_{p'} A(f\mid p')\right) \\ c^{(t+1)}&= \arg \max _c \sum _{p\in {\mathcal {P}}} \left( \sum _{f\in F} z^{(t)}_{f, p}\right) \log c_p, \quad \text {s.t.} \sum _{p\in {\mathcal {P}}} c_p {\hat{l}}_p = 1 \end{aligned}$$

$z^{(t)}$ and $c^{(t)}$ denote the variables at iteration *t*. We hide the constraint from prefix graphs for clarity. The optimization for $z^{(t)}_{f, p}$ can be run in parallel, so we focus on the M-step that optimizes $c^{(t+1)} = \{c^{(t+1)}_p\}$, hiding the superscript whenever it is clear from context. When we optimize over the whole genome, the instance becomes impractically huge. This is because we need to infer the flow for every prefix graph (one for each gene) across the whole genome, and we need to satisfy flow balance for each graph and normalization for all graphs together.

We let ${\mathcal {G}}$ denote the set of genes. Denote the gene abundance $c_g = \sum _{p\in {\mathcal {P}}_g} c_p{\hat{l}}_p$, where ${\mathcal {P}}_g$ is the set of phasing paths in gene *g*. We then define relative abundance $c^*_p = c_p / c_g$ for every phasing path *p*. Plugging $c_p = c_g c^*_p$ into the expression for M-step, we have the following transformed objective:

$$\begin{aligned} \max &\quad \sum\limits_{{p \in {\mathcal{P}}}} {\left( {\sum\limits_{{f \in F}} {z_{{f,p}}^{{(t)}} } } \right)} (\log c_{p}^{*} + \log c_{g} ) \hfill \\ \; \quad \quad &= \sum\limits_{{g \in {\mathcal{G}}}} {\sum\limits_{{p \in {\mathcal{P}}_{g} }} {\left( {\sum\limits_{{f \in F}} {z_{{f,p}}^{{(t)}} } } \right)} } \log c_{p}^{*} + \sum\limits_{{g \in {\mathcal{G}}}} {\left( {\sum\limits_{{f \in F}} {\sum\limits_{{p \in {\mathcal{P}}_{g} }} {z_{{f,p}}^{{(t)}} } } } \right)} \log c_{g} \hfill \\ \quad \quad & = \sum\limits_{{g \in {\mathcal{G}}}} {\sum\limits_{{p \in {\mathcal{P}}_{g} }} {\left( {\sum\limits_{{f \in F}} {z_{{f,p}}^{{(t)}} } } \right)} } \log c_{p}^{*} + \sum\limits_{{g \in {\mathcal{G}}}} {s_{g} } \log c_{g} \hfill \\ {\text{s.t.}}& \quad \sum\limits_{{p \in {\mathcal{P}}}} {c_{g} } c_{p}^{*} \hat{l}_{p} = \sum\limits_{{g \in {\mathcal{G}}}} {c_{g} } = 1 \hfill \\ & \quad \sum\limits_{{p \in {\mathcal{P}}_{g} }} {c_{p}^{*} } \hat{l}_{p} = 1,\forall g \in {\mathcal{G}} \hfill \\ \end{aligned}$$

Here, $s_g = \sum _{f\in F} \sum _{p\in {\mathcal {P}}_g} z^{(t)}_{f, p}$ can be interpreted as the estimated read count of gene *g*. Again for clarity, we hide the prefix graph constraints for $c^*_p$, which retain their original form because all prefix graph constraints are affine. Now, we can decouple optimization of $c^*_p$ and $c_g$, as the objective function is split into two parts, and each constraint only involves one of them. The optimization for $c^*_p$ can be done for each gene independently, and it is exactly the single-gene optimization as we described above except we weight $\log c_{p(f)}$ with $z^{(t)}_{f, p}$. The optimization for $c_g$ has the form $\max \sum _{g\in {\mathcal {G}}} s_g \log c_g$ constrained by $\sum _{g\in {\mathcal {G}}} c_g = 1$, from which we derive that $c_g \propto s_g$. Since $\sum _{g\in {\mathcal {G}}} s_g = \sum _{p\in {\mathcal {P}}} \sum _{f\in F} z^{(t)}_{f, p} = \sum _{f\in F} 1 = |F|$, we have the following localized EM algorithm:

$$\begin{aligned} \text {Global E-step:} \ \ \ z^{(t)}_{f, p}&= c^{(t)}_pA(f\mid p) / \left( \sum _{p'\in M(f)} c^{(t)}_{p'} A(f\mid p')\right) \\ \text {Gene-Level M-step:} \ \ \ c^{(t+1)}&= \arg \max _c \sum _{p\in {\mathcal {P}}_g} \left( \sum _{f\in F} z^{(t)}_{f, p}\right) \log c_p \\&\text {s.t.} \sum _{p\in {\mathcal {P}}_g} c_p{\hat{l}}_p = \sum _{p\in {\mathcal {P}}_g}\sum _{f\in F} z^{(t)}_{f,p} / |F|, \forall g\in {\mathcal {G}} \end{aligned}$$

The M-step is run independently for each gene and can be parallelized. We do not list the prefix graph constraint over $c_p$ for clarity, and $c_g$ is implicitly derived as the right-hand side of the normalization constraint.

## Experiments

Based on the expression quantification method Salmon [3] and its effective lengths, we implement our method and call it Graph Salmon. We apply Graph Salmon on three bipolar disease (BD) RNA-seq samples and three control samples [18] to estimate the expression network flow on neurogenesis-related genes (GO:0022008), which are known to have complex alternative splicing patterns and novel isoforms. We use this as a case study to show that Graph Salmon is applicable with variable fragment lengths and that the relative usage of splice junctions under the incomplete reference assumption are different from those under complete reference assumption.

### Implementation

The splice graphs are constructed using the reference exons and splice junctions of Gencode [19] version 26. Since Salmon’s effective lengths are needed for path effective lengths, we first run Salmon on the samples. We also use Salmon read mappings (obtained with the –writeMappings argument) and convert their coordinates onto splice graph nodes and edges. Prefix graphs are constructed with the converted read mappings. Each edge in the prefix graph corresponds to a path in the original splice graph, and we compute the path effective length by taking the average of the effective lengths of the corresponding region in reference transcripts that include the corresponding path (for detail see Additional file 1: Section 1.1). With the converted read mappings and path effective lengths, the probabilistic model of graph quantification can be specified.

Since only neurogenesis-related genes are of interest and the rest of the genes are assumed to have complete reference transcripts, we assume that Salmon correctly estimates the probability of each paired-end read generated from each gene when the read is mapped to multiple genes. We use Salmon’s gene-level weight assignment as the read counts and only solve the flow optimization problem within each gene, which corresponds to one round of the gene-level M step for each gene.

### Graph Salmon reveals unique between-sample differences of PSI for neurogenesis genes

The mean fragment lengths of the six samples range from 349.17 to 375.28 bp. The standard deviations of fragment lengths are between 53.00 and 82.15 bp. Meanwhile, 30% of the exons (or subexons) across the splice graphs are less than 56 bp long, and the 40% quantile of subexon lengths is 79 bp. Graph Salmon is needed in this dataset because of the large standard deviation of fragment lengths compared to subexon lengths.

We computed Percentage Spliced In (PSI) of 2441 skipped exon events using the Graph Salmon network flow and compare them with PSIs calculated using Salmon’s expression quantification based on the reference transcripts. Given three exons, the PSI is defined as the total abundance of transcripts that include all three of them, divided by total abundance of transcripts that include the first and the last (but not necessarily the middle one).

The correlations of Graph Salmon PSI and Salmon PSI of the same sample are around 0.51 to 0.57 (for both Spearman and Pearson), while the correlations of PSI between different samples computed by the same quantification method are over 0.75 (for both Spearman and Pearson and both methods). The large correlation between different samples can be explained by the fact that they are from the same tissue and should follow the tissue-specific expression and alternative splicing patterns. The smaller correlation between different quantification methods indicates the incomplete reference and complete reference assumptions lead to very different splice junction abundance estimates.

**Fig. 2:**
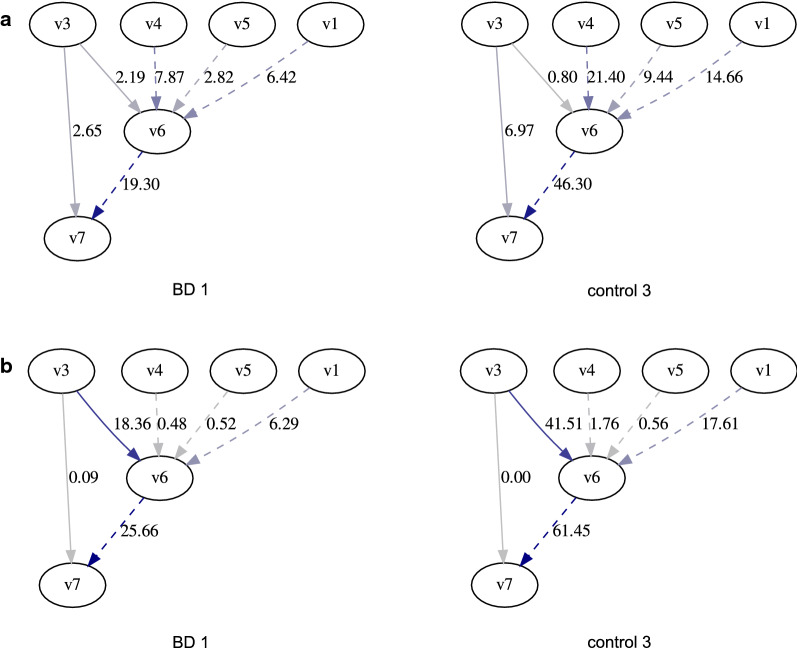
Graph Salmon and Salmon give different PSI estimates in an example of BD RNA-seq sample. **a** Network flow of BD 1 and control 3 samples estimated by Graph Salmon. The subgraph includes exons 1, 3 to 7, and exons are represented by nodes and the node label indicates the index of exon. PSI of inclusion of exon 6 between exon 3 and 7 is computed. Edges involved in the PSI calculation are solid; the rest are dashed. **b** Network flow of the same samples computed by Salmon with reference transcripts

An example of different PSI computed by Graph Salmon and Salmon is shown in Figure 2 and Additional file 1: Figure S2 on *LPAR1* gene. *LPAR1* gene encodes a lysophosphatidic acid (LPA) receptor that functions in the LPA signaling pathway, which is related to cognitive behavioral deficits such as schizophrenia and depression when dysregulated [20]. We focus on the event that describes the percentage of expression of the inclusions of exon 6 (position 110973480–110973558 in GRCh38) between exon 3 (position 111037840–111038043 in GRCh38) and exon 7 (position 110972072–110972220 in GRCh38). Graph Salmon computes the PSIs to be 0.45 to 0.64 for BD samples and 0.07 to 0.33 for control samples, whereas PSIs computed by Salmon are larger than 0.95 for all six samples.

Even though this difference is not evaluated by rigorous statistical testing, it indicates that when the reference is incomplete, previous reference-based alternative splicing analysis may lead to different results. Considering the incomplete reference assumption in alternative splicing analysis enlarges the pool of candidate alternative splicing events.

### Evaluation the prefix graph size with increase of read length and sequencing coverage

Even though the prefix graph is able to encode the phasing information of variable-length fragments, the size of prefix graph can greatly increase to a degree such that inference on prefix graph becomes a computational burden. We evaluate the size of the prefix graph under several ranges of read lengths and sequencing coverages by simulated RNA-seq data.

The simulation setup is as follows. Since the splice graph and prefix graph is constructed for each gene, we select a subset of genes for simulating RNA-seq reads and evaluating prefix graph sizes for computational efficiency. 500 genes are randomly selected under the condition that they are multi-isoform genes and the total number of $S-T$ paths in their splice graphs exceed 10. We use the reference transcripts as the set of expressed transcripts from the randomly selected genes. Paired-end RNA-seq reads are simulated by polyester [21] under one of the various sequencing coverage settings (50X, 200X coverage) and under one of the read length settings (100 bp, 300 bp, 500 bp). The length of sequenced fragments is twice the read length plus the insert size. Graph Salmon is applied to the simulated RNA-seq dataset as described in Section 3.1. We use the edge count of the prefix graph of each gene to evaluate the size of prefix graph because the number of prefix graph edges is the number of parameters to be inferred in graph quantification.

**Fig. 3:**
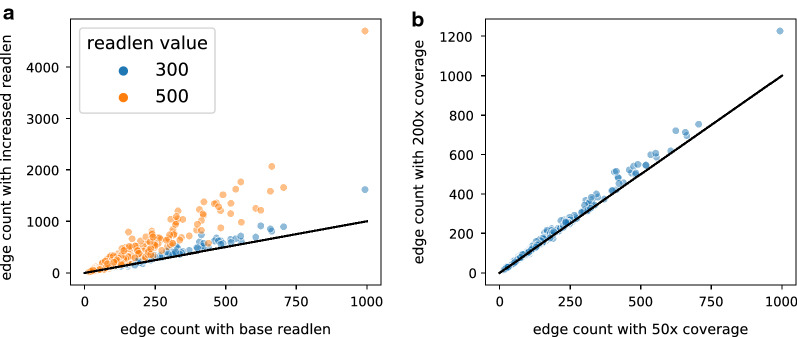
Size increase of prefix graph under different read lengths and sequencing coverages. **a** Scatter plot between the prefix graph edge count under base read length (100 bp) and that under an increased read length (300 bp and 500 bp). **b** Scatter plot between the prefix graph edge count under 50X sequencing coverage and that under 200X sequencing coverage

With increased read lengths, the number of prefix graph edges greatly increased as shown in Fig. 3a. But the edge counts for most of the genes occupy the smaller end of the figure axes, and the genes with extremely large number of prefix graph edges are rare. We also observe that with a fixed number of expressed isoforms, the rate of edge count increase is not as fast as an exponential growth.

The prefix graph edge counts do not increase a lot with the increase of sequencing coverage (Fig. 3b). This is expected since a larger coverage increases the probability that a phasing path in splice graph is captured by some sequencing fragment, but the total number of possible phasing paths is bounded by the splice graph structure and the read length. Overall, these results indicate the prefix graph for graph quantification can be applied to next-generation RNA-seq data under various coverage; but the computation is impractical when applied to third-generation RNA-seq with read lengths of several thousand base pairs.

## Conclusion

We improve the graph quantification model of FlipFlop to incorporate phasing information from variable length reads or fragments. The key algorithmic contributions are a provably correct reparameterization process and the introduction of the prefix graph inspired by Aho-Corasick automata for inference.

To demonstrate the feasibility of our method to handle variable length fragments, we apply our method to neurogenesis-related genes of bipolar disease RNA-seq samples and control RNA-seq samples. The RNA-seq samples contain paired-end reads with mean fragment lengths around 350 bp and standard deviation around 53–82 bp. We show that our method successfully estimates network flows on prefix graphs and the estimated flow (under the incomplete reference assumption) only has around 0.5 correlation (both Pearson and Spearman) with the flow estimated by Salmon under the complete reference assumption.

The size of the prefix graph depends on the length of the phasing paths exponentially. Unfortunately, for long-read sequencing, especially with transcript-long reads, the prefix graph may be as large as the set of all $S-T$ paths (equivalently the set of all possible transcripts) and its efficiency compared to the naïve implementation of graph quantification (where we enumerate every compatible transcript) may diminish. It is still open what algorithmic tools are required to avoid this inefficency.

An intrinsic issue with graph quantification is non-identifiability: Many configurations of transcript abundances lead to the same read generation model, and thus it is impossible to distinguish which configuration is closer to the ground truth if our goal is to recover an underlying transcriptome. While our prefix graph representation is compact, for many downstream analyses, we are invariably forced to perform a flow decomposition to transform prefix graph flow into quantified transcript sets. The non-identifiability problem manifests in this step, as different decompositions can lead to the same prefix graph flow, which as we proved implies the same model of read generation. Therefore, it is possible to assess the severity of non-identifiability problem by inspecting different ways of decomposing a fixed prefix graph flow.

This work focuses on theoretical improvements of the graph quantification model, while its practical utility is still largely unexplored. For example, our proposed approach may be a promising method for transcript assembly similar to FlipFlop, where we use quantification for assembly. The method also has potential use cases in alternative splicing analyses and other related tasks in RNA-seq. However, careful benchmarking is needed to determine the cases when graph quantification is superior than standard quantification with a given set of transcripts for each task.

## Supplementary Information

**Additional file 1.** More details on FlipFlop algorithm. This additional file provides more explanation on the algorithm of FlipFlop and why it cannot handle variable-length sequencing fragments. It also includes the figure showing the edge abundance estimation of LPAR1 gene in the other BD and control samples.

## Data Availability

The source code of Graph Salmon is available at https://github.com/Kingsford-Group/subgraphquant. The RNA-seq sampled used in this study can be accessed from Gene Expression Omnibus (GEO) database with accession numbers GSM1288369, GSM1288370, GSM1288371 for bipolar disease samples, and GSM1288374, GSM1288375, GSM1288376 for control samples.

## Abbreviations

RNA-seq: RNA sequencing
PSI: Percentage Spliced In
EM: Expectation-maximization
TPM: Transcripts per Million
GC-content: Guanine-cytosine content
FSA: Finite state automaton
DAG: Directed acyclic graph
BD: Bipolar disease
GEO: Gene expression omnibus
LPA: Lysophosphatidic acid
GO: Gene ontology
GRCh38: Genome Reference Consortium Human Build 38
