# Supplementary material for: Exact transcript quantification over splice graphs
Source: Algorithms Mol Biol. 2021 May 10;16:5. doi: 10.1186/s13015-021-00184-7 (PMC8112020; doi:10.1186/s13015-021-00184-7)
Supplement: Supplementary file 1 — Additional file 1. More details on FlipFlop algorithm. This additional file provides more explanation on the algorithm of FlipFlop and why it cannot handle variable-length sequencing fragments. It also includes the figure showing the edge abundance estimation of LPAR1 gene in the other BD and control samples. [file 13015_2021_184_MOESM1_ESM.pdf]

Exact Transcript Quantification over Splice Graphs
Additional file 1

Cong Ma*[1], Hongyu Zheng*[2], and Carl Kingsford†[2]

[1]Computer Science Department, School of Engineering and Applied Science, Princeton University, 35 Olden St., Princeton, NJ

[2]Computational Biology Department, School of Computer Science, Carnegie Mellon University, 5000 Forbes Ave., Pittsburgh, PA

January 2021

1 Supplementary Methods

1.1 FlipFlop and the Fragment Graph

The fragment graph constructed by FlipFlop is defined as follows. Given splice graph G and a set of phasing paths $\mathcal{P}$ (again we consider a general notion of phasing paths, meaning single exon paths also count as phasing), the fragment graph G_F is constructed such that

- Each vertex in G_F is either S, T, or a phasing path.
- There is an edge connecting X to Y only if Y is a single exon extension or shrinking of X (unless one of them is either S or T).
- Every $S-T$ path in the splice graph can be mapped uniquely to a $S-T$ path in the fragment graph, and the set of vertices included in the $S-T$ path is exactly the set of phasing paths that are a subpath of the transcript.

We only list a necessary condition in the second item, and we will not discuss how the graph is constructed in practice here. The third item is essential for the FlipFlop algorithm, as it solves the inference problem with convex cost flows which requires that every phasing path is represented by a single vertex in the graph.

As discussed in the introduction, the FlipFlop algorithm is correct when input library is single-end reads with fixed read length. This implies that if X is a phasing path, there are no phasing paths that are extension of X on both ends (for example, if $X = [3, 4]$, then $[2, 3, 4, 5]$ cannot be a phasing path), otherwise it would violate the condition that all reads have equal length. We now show that there exists no correct fragment graph when the condition is violated.

*Equal contribution This work was done when C. M. was a Ph.D. student at Carnegie Mellon University.
†To whom correspondence should be addressed: carlk@cs.cmu.edu

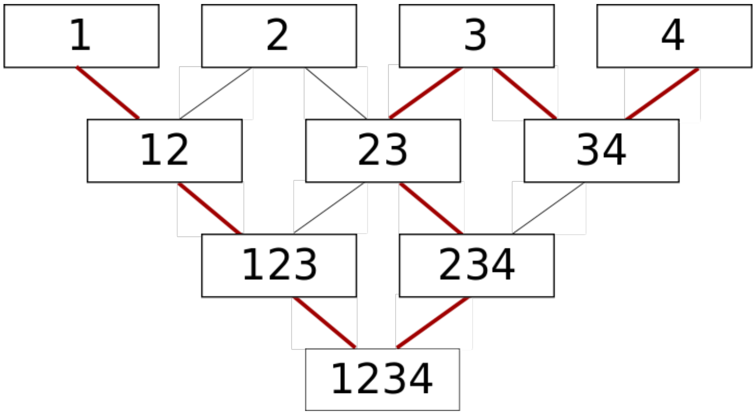

Figure S1: The fragment graph with 4 exons and 10 phasing paths, not including S and T. Blocks denote vertices of the fragment graph, and lines denote possible edges between vertices (phasing paths). A path visiting 9 vertices (excluding the singleton phasing path $[2]$) is marked in dark red, and there is no Hamiltonian path in the graph.

Consider a splice graph with a chain of four exons denoted $1, 2, 3$ and 4, and where every subpath of $[1, 2, 3, 4]$ is a phasing path. The fragment graph, if exists, will contain 10 vertices (excluding S and T) and a Hamiltonian path corresponding to the transcript $[1, 2, 3, 4]$. However, as seen in the above figure, the graph will not contain a Hamiltonian path no matter how the graph is constructed.

2 Supplementary Figure

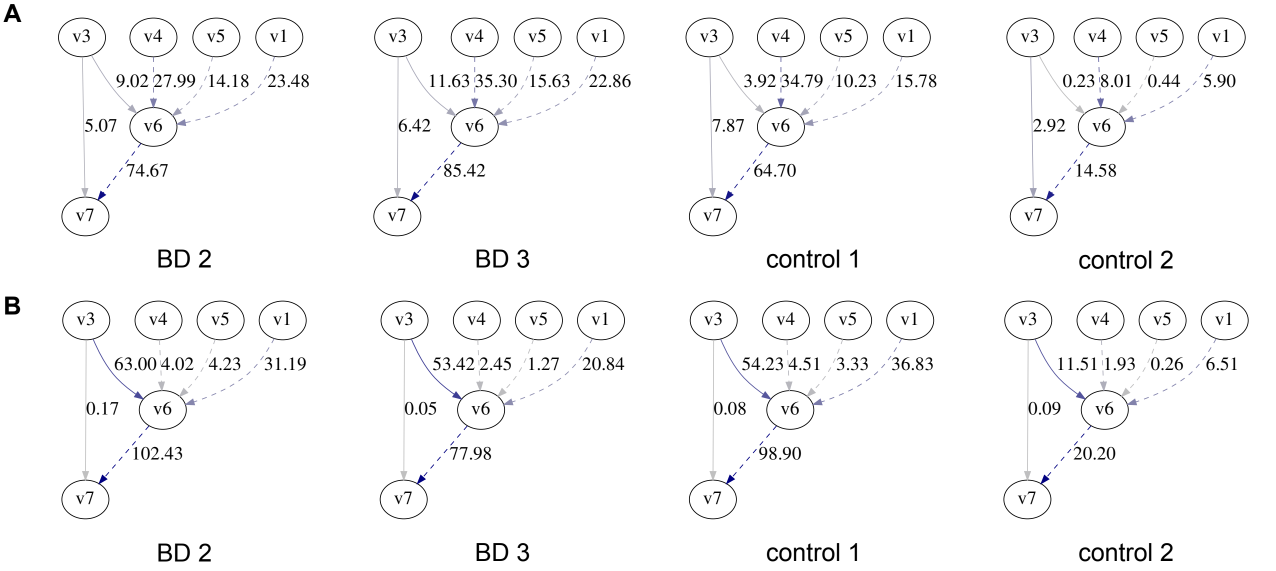

Figure S2: **Splice edge abundances estimation of the rest four BD RNA-seq sample.** (A) Network flow of BD 2, BD3, control 1, and control 2 samples estimated by Graph Salmon. The subgraph includes exons 1, 3 to 7, and exons are represented by nodes and node label indicates the index of exon. PSI of inclusion of exon 6 between exon 3 and 7 is computed. Edges of which the flows are involved in PSI calculation are solid; the rest edges are dashed. (B) Network flow of the same samples computed by Salmon with reference transcripts.
